# *Labisia pumila* regulates bone-related genes expressions in postmenopausal osteoporosis model

**DOI:** 10.1186/1472-6882-13-217

**Published:** 2013-09-05

**Authors:** Siti Noor Fathilah, Norazlina Mohamed, Norliza Muhammad, Isa Naina Mohamed, Ima Nirwana Soelaiman, Ahmad Nazrun Shuid

**Affiliations:** 1 Department of Pharmacology, Faculty of Medicine, The National University of Malaysia (UKM), Jalan Raja Muda Abd Aziz, 50300, Kuala Lumpur, Malaysia; 2 Division of Pharmacology, Department of Human Anatomy, Faculty of Medicine and Health Sciences, Universiti Putra Malaysia, 43400, UPM Serdang, Selangor, Malaysia

**Keywords:** *Labisia pumila*, Postmenopausal osteoporosis, Estrogen, OPG, BMP-2, RANKL, MCSF

## Abstract

**Background:**

*Labisia Pumila var. alata* (LPva) has shown potential as an alternative to estrogen replacement therapy (ERT) in prevention of estrogen-deficient osteoporosis. In earlier studies using postmenopausal model, LPva was able to reverse the ovariectomy-induced changes in biochemical markers, bone calcium, bone histomorphometric parameters and biomechanical strength. The mechanism behind these protective effects is unclear but LPva may have regulated factors that regulate bone remodeling. The aim of this study is to determine the bone-protective mechanism of LPva by measuring the expressions of several factors involved in bone formative and resorptive activities namely Osteoprotegerin (OPG), Receptor Activator of Nuclear Factor kappa-B Ligand (RANKL), Macrophage-Colony Stimulating Factor (MCSF) and Bone Morphogenetic Protein-2 (BMP-2).

**Methods:**

Thirty-two female Wistar rats were randomly divided into four groups: Sham-operated (Sham), ovariectomized control (OVXC), ovariectomized with *Labisia pumila var. alata* (LPva) and ovariectomized with ERT (Premarin®) (ERT). The LPva and ERT were administered via daily oral gavages at doses of 17.5 mg/kg and 64.5 μg/kg, respectively. Following two months of treatment, the rats were euthanized and the gene expressions of BMP-2, OPG, RANKL and MCSF in the femoral bones were measured using a branch - DNA technique.

**Results:**

The RANKL gene expression was increased while the OPG and BMP-2 gene expressions were reduced in the OVXC group compared to the SHAM group. There were no significant changes in the MCSF gene expressions among the groups. Treatment with either LPva or ERT was able to prevent these ovariectomy-induced changes in the gene expressions in ovariectomized rats with similar efficacy.

**Conclusion:**

LPva may protect bone against estrogen deficiency-induced changes by regulating the RANKL, OPG and BMP-2 gene expressions.

## Background

Osteoporosis is defined as a systemic skeletal disease that is characterized by low bone mass and micro-architectural deterioration of bone tissue, with a consequent increase in bone fragility and susceptibility to fracture [1]. According to the World Health Organization [2], osteoporosis occurs when the bone mineral density falls more than 2.5 standard deviations (SD) below the standard reference for maximum bone mineral density of young adult females. The bone mass in females begins to decline slowly after the age of 35 to 40, followed by a phase of dramatic bone loss after menopause. This is due to estrogen deficiency which occurs naturally with aging or by surgical ovariectomy.

By 50 years of age, the bone mass in women is only two-thirds of that in men [3]. The relatively lower bone mass in women is due to a combination of lower peak bone mass and faster rate of bone loss. This leads to higher incidence of osteoporosis in elderly women compared to men [4]. Osteoporosis should be managed appropriately as statistics have shown that 1 in 3 women aged more than 50 years old suffered an osteoporotic fracture during their lifetime [5].

Estrogen Replacement Therapy (ERT) is one of the main form of treatment and prevention of postmenopausal osteoporosis. Estrogen given alone or in combination with progesterone is able to prevent postmenopausal osteoporosis effectively [6]. Estrogen binds to its receptors on the osteoclast surface, causing the release of chemical mediators which led to reduction of osteoclastic activity and inhibition of bone resorption [7].

The “Women’s Health Initiative Study” found that women who took ERT have slightly higher rates of ovarian cancer, breast cancer, heart attack, thromboembolism, stroke, and Alzheimer’s disease [8-10]. These negative reports on the serious adverse-effects of ERT have led to many postmenopausal women searching for available alternatives for their postmenopausal symptoms. These have also paved way for missions to discover alternative anti-osteoporotic agents that are comparable in effectiveness to estrogen but with minimal adverse effects. Several potential alternative agents were discovered including soy [11], blueberry [12], *Achyranthes bidentata*[13] and tocotrienol [14]. Recently, *Labisia pumila var. alata* (LPva), a herb used for women’s health, was found to produce beneficial effects similar to estrogen on bone biomarkers in postmenopausal osteoporosis animal model [15]. These bone protective effects were further confirmed by bone histomorphometric analysis [14-16] and bone biomechanical strength [15-17].

*Labisia Pumila* (LP), a plant from the family of *Myrinaceae* is a popular herb in Malaysia, Indonesia and Indo-China. There are three variants of *Labisia pumila*, which are *var. alata* (LPva), *var. pumila* and *var. lanceolata*[18]. In Malaysia, it is known locally as “Kacip Fatimah” and the extract can simply be prepared by boiling the leaves, roots or the whole plant in water and the extract is taken orally [18,19]. Nowadays, various *Labisia pumila* preparations are available commercially in the forms of capsules or added to drinks. It is used exclusively by women to shrink the uterus, facilitate labor, and improve menstrual irregularities and as post-partum medicine [19,20]. Its limited use by women for their health supplements has led to the belief that it is a phytoestrogen, a compound with similar chemical structure to estrogen [21]. Several studies have demonstrated the estrogenic properties of LPva. It was found to inhibit estradiol binding to antibodies raised against estradiol [22] and exert a specific estrogenic effect on human endometrial adenocarcinoma cells (Ishikawa-Var I line) [23]. It mimicked estrogen action by preventing the shrinkage of the uterus due to estrogen deficiency in ovariectomized rats [22] and initiate lipolysis in adipose tissue [24]. LPva was also found to down-regulate 11β-hydroxysteroid dehydrogenase-1 expressions in liver and adipose tissues and also decrease serum corticosterone levels in ovariectomized rats [25]. In terms of bone protection against estrogen deficiency, LP was reported to exert estrogen-like effects on bone remodeling [15-17].

Bone remodeling involves a fine balance between bone formation and resorption. Any disturbance to the balance between these two processes would lead to bone pathology. There are several factors or cytokines known to play important roles in bone remodeling. Bone resorption is regulated by Receptor Activator of Nuclear Factor kappa-B ligand (RANKL) and Osteoprotegerin (OPG), which are produced by osteoblasts [26]. RANKL binds to RANK receptors which are located on osteoclast precursors to promote differentiation into mature osteoclasts and activate their bone resorptive activity [27]. It was reported that administration of serum RANKL to mice promoted osteoclast growth and activation, leading to osteoporosis [28]. OPG acts as anti-resorptive decoy receptor by binding to RANKL and preventing it from binding to RANK receptors. As a result, OPG inhibits osteoclast differentiation and its bone resorptive activity. In a recent development, a fully human monoclonal antibody against RANKL was developed to inhibit osteoclast activity. This new agent named denosumab is still under clinical trial as a new anti-osteoporotic agent [29].

Osteoclastogenesis also requires Macrophage-Colony Stimulating Factor (MCSF), which is also expressed by osteoblasts. It binds to the MCSF receptors situated in the osteoclasts and stimulates osteoclastogenesis, but the mechanism involved is still unclear [30]. There are several factors which are known to affect osteoblast activity and differentiation. Among the important one is bone morphogenetic protein-2 (BMP-2), which promotes osteoblast differentiation and plays an important role in bone repair and regeneration [31]. In summary, the differentiation and activation of osteoclast are influenced by the RANKL/OPG system and MCSF, while BMP-2 plays a key role in osteoblast differentiation. Estrogen was able to maintain bone density by regulating the gene expressions of these factors [32] and LPva may have similar actions. To the best of our knowledge, there is no study on the mechanism of LPva extract in preventing bone loss due to estrogen deficiency. Therefore, the aim of the study is to determine the molecular mechanism of LPva in protecting bone against estrogen-deficient osteoporosis by measuring the bone-related gene expressions.

## Methods

### Animal and treatment

Thirty-two female Wistar rats, aged three months old and weighing 200 to 250 grams, were used as the animal model in this study. The rats were allowed to acclimatize for a week before the start of the study. They were housed two per cage, at normal room temperature with adequate ventilation and normal 12-hour light-dark cycle. All rats were allowed free access to water and food (commercial laboratory rat’s food containing 0.97% calcium, 0.85% phosphorus and 1.05 IU/g of Vitamin D3)(Gold Coin, Selangor, Malaysia). They were randomly divided into four main groups. The sham-operated group (Sham) and the ovariectomized control group (OVXC) were given oral gavages of deionized water (vehicle) throughout the study. The treatment group was either given LPva at the dose of 17.5 mg/kg (LPva group) or Premarin® at the dose of 64.5 μg/kg (ERT group) via daily oral gavages for the duration of 8 weeks. The ERT group acted as the positive control group. The rats were euthanized upon completion of the study and both femora dissected out and cleaned of any tissues. The approval for this study was obtained from the University Animal Ethic Committee of Universiti Kebangsaan Malaysia (PP/FAR/2009/NAZRUN/14 JULY/267-JULY 2009-MAY-2010).

### *Labisia pumila var. alata (LP)* extract

The root extract of LPva (Batch No: KF071107) was purchased from Phytes Biotek Sdn Bhd. (Malaysia), a Good Manufacturing Practice (GMP) licensed manufacturer of herbal products, in the form of a freeze-dried standardized extract. All LPva extractions were performed at the factory in Shah Alam, Selangor, Malaysia, using a patented high-pressure water extraction process (US 7,132,117 B2). They were filtered at 1 to 4 mm and freeze-dried without maltodextrin or lactose. The brownish powdered LPva extract used in this study was similar to the extract used in previous studies by Fathilah *et al.*[16,17] and Nazrun *et al.*[15]. This extract was also the same form used for human consumption as health supplements. Phytochemical testing of the LPva extract by the Forest Research Institute Malaysia (FRIM) found the extract to contain flavonoids, saponins and triterpenes. The LPva extract was dissolved in deionised water and given to the LPva group via oral gavages at the dose of 17.5 mg/kg rat weight at 9 am daily for 8 weeks [15-17]. The Premarin® (Wyeth-Ayerst, Canada) tablet containing 0.625 mg of conjugated estrogen was crushed, dissolved in deionised water and given to the ERT group via oral gavages at the dose of 64.5 μg/kg rat weight at 9 am daily for 8 weeks [15-17]. These doses were chosen based from our previous studies, which have demonstrated protective effects against estrogen-deficient osteoporosis [15-17].

### Gene expression measurements

Gene expressions were measured using branched DNA (bDNA) technique as described by Fathilah *et. al*[33]. The bDNA assay is a sandwich nucleic acid hybridization method that uses bDNA molecules to amplify signals from captured target RNA. Briefly, the femoral bones were ground into a fine powder with a pestle and mortar with the addition of liquid nitrogen. Proteinase K was added to release the ribonucleic acid (RNA), which was then prepared according to directions suggested by Panomics (Fremont, CA) for analysis of mRNA expression using the Panomics QuantiGene Plex 2.0 systems. The bDNA method combines RNA signal amplification and microspheres with unique fluorescent signatures to enable quantitation of multiple mRNA targets simultaneously in the same sample, without having the amplification inaccuracies of RT-PCR (reverse transcriptase–polymerase chain reaction). The method allows for discrimination of highly homologous messages [34,35]. Specific oligonucleotide capture and extender probe sets (3 per target) annealed exclusively to each mRNA of interest and the housekeeping mRNAs. They were designed according to the unique sequences within each message sequence. The housekeeping genes used in this study were glyceraldehyde-3-phosphate dehydrogenase (GAPDH), glucuronidase-beta (Gusb) and hypoxanthine phosphoribosyltransferase-1 (HPRT1). Specific mRNA transcripts were captured to specific fluorescent beads by hybridization to capture probe-extender probe interactions. The signal from each hybridized unit was amplified by attachment of biotinylated label probes at multiple binding sites on the complexes, which were in turn bound to streptavidin-conjugated R-phycoerythrin (SAPE) to produce fluorescence. The fluorescent signals associated with individual capture beads were read using a Luminex100 IS system (Luminex Corp., Austin, TX, US) with the bead signature designating RNA target and the SAPE signal designating abundance. For each well, the total fluorescence from each individual bead type (corresponding to individual mRNA species) minus the background fluorescence for that bead type was normalized to the geometric mean of the fluorescence of the 3 housekeeping genes. The normalized signals for individual mRNAs from triplicate wells were averaged to yield a single value for each mRNA species being measured.

### Statistical analysis

The results were expressed as mean ± standard error of the mean (SEM). The data analysis was performed using the Statistical Package for Social Sciences software (SPSS 19; SPSS, Chicago, IL, USA). The data were first tested for normality using the Kolmogorov–Smirnov test. For normally distributed data, the statistical tests used were the analysis of variance (ANOVA), followed by Tukey’s Honestly Significant Difference (HSD) test. For data that were not normally distributed, Kruskal–Wallis and Mann–Whitney tests were used.

## Results

### RANKL gene expressions

**Figure 1:**
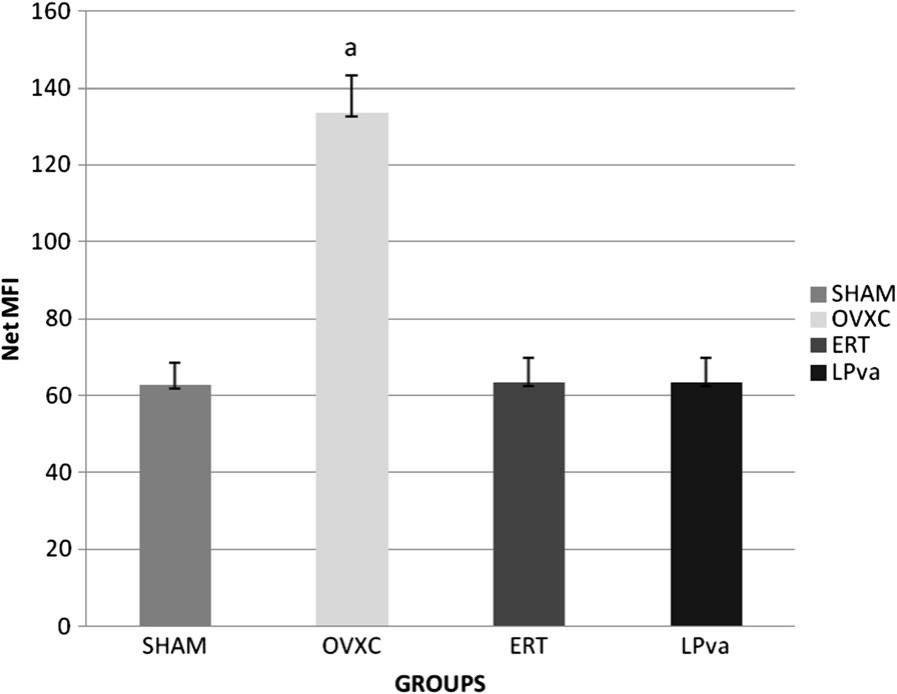
**RANKL Gene Expression.** Samples were normalized to GAPD MFI (MedianFluorescence Intensity). Error bars represent the standard deviations of the average responses. Values were expressed as mean ± SEM*; p < 0.05* is considered significant. ^a^ indicates significant difference compared to the Sham group. Sham (water vehicle), OVXC (water vehicle), LPva (*Labisia pumila var. Alata* 17.5 mg/kg/day), ERT (Premarin 64.5 μg/kg/day).

The RANKL gene expression of the femoral bones was significantly higher in the OVXC group compared to other groups. Treatment with ERT was able to prevent the ovariectomy-induced elevation of RANKL gene expression, until it was at the same level as the Sham group. LPva supplementation was also able to prevent the ovariectomy-induced elevation of RANKL gene expression. There were no significant differences in the RANKL gene expression levels between the Sham, ERT and LPva groups (Figure 1).

### OPG gene expressions

**Figure 2:**
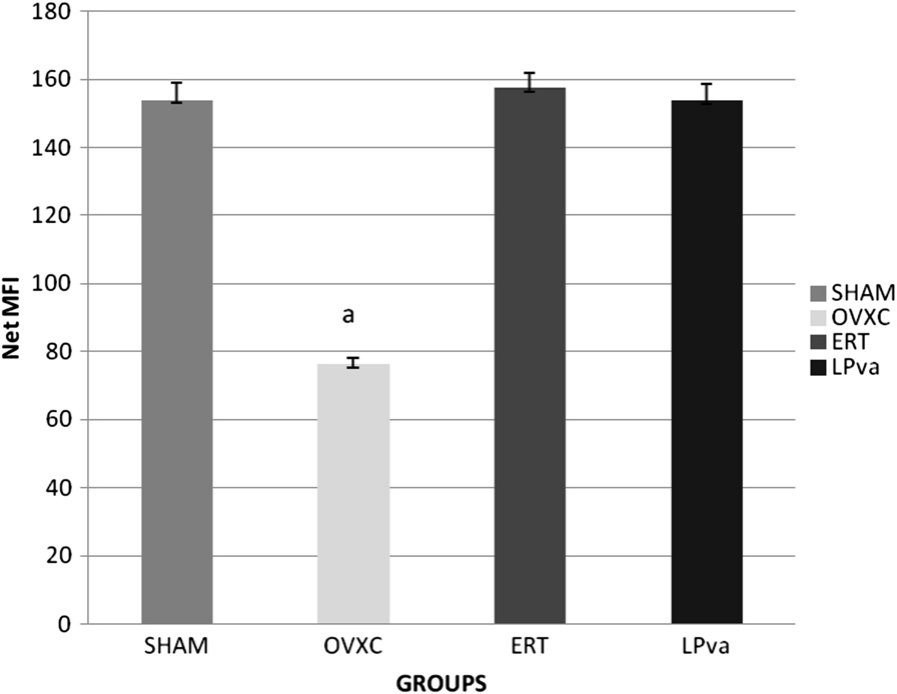
**Osteoprotegerin Gene Expression.** Samples were normalized to GAPD MFI (MedianFluorescence Intensity). Error bars represent the standard deviations of the average responses. Values were as mean ± SEM*; p < 0.05* is considered significant. ^a^ indicates significant difference compared to the Sham group. Sham (water vehicle), OVXC (water vehicle), LPva (*Labisia pumila var. Alata* 17.5 mg/kg/day), ERT (Premarin 64.5 μg/kg/day).

The OPG gene expression of the femoral bones was significantly lower in the OVXC group compared to the other groups. Treatment with ERT was able to prevent the ovariectomy-induced reduction of OPG gene expression, until it was at the same level as the Sham group. LPva supplementation was also able to prevent the ovariectomy-induced reduction of OPG gene expression. There were no significant differences in the OPG gene expression levels between the Sham, ERT and LPva groups (Figure 2).

### MCSF gene expression

**Figure 3:**
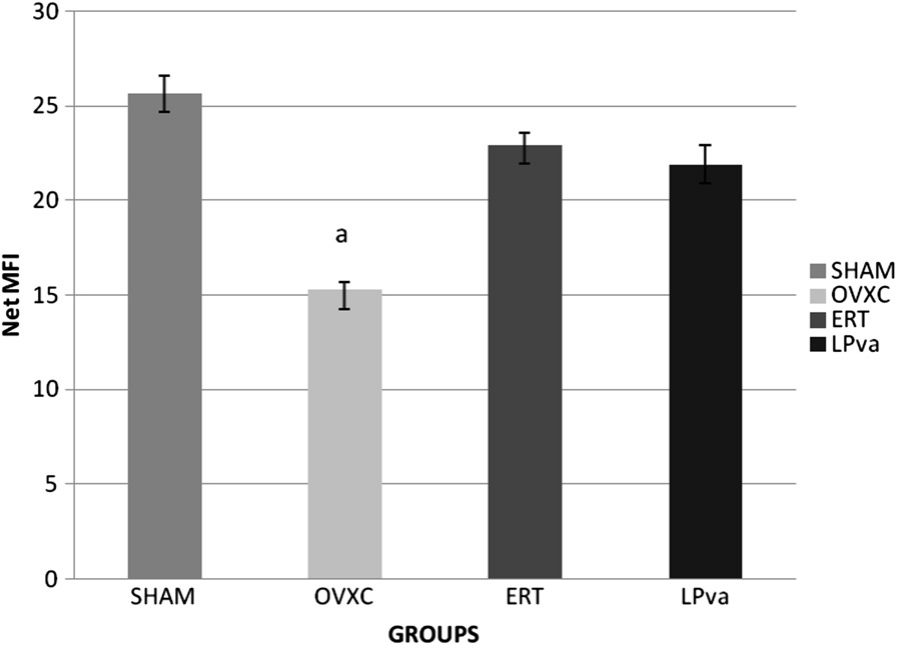
**BMP-2 gene expression.** Samples were normalized to GAPD MFI (MedianFluorescence Intensity). Error bars represent the standard deviations of the average responses. Values were as mean ± SEM; *p < 0.05* is considered significant. ^a^ indicates significant difference compared to the Sham group. Sham (water vehicle), OVXC (water vehicle), LPva (*Labisia pumila var. Alata* 17.5 mg/kg/day), ERT (Premarin 64.5 μg/kg/day).

There were no significant changes in MCSF gene expressions of the femoral bones for all the groups (Figure 3).

### BMP-2 gene expression

**Figure 4:**
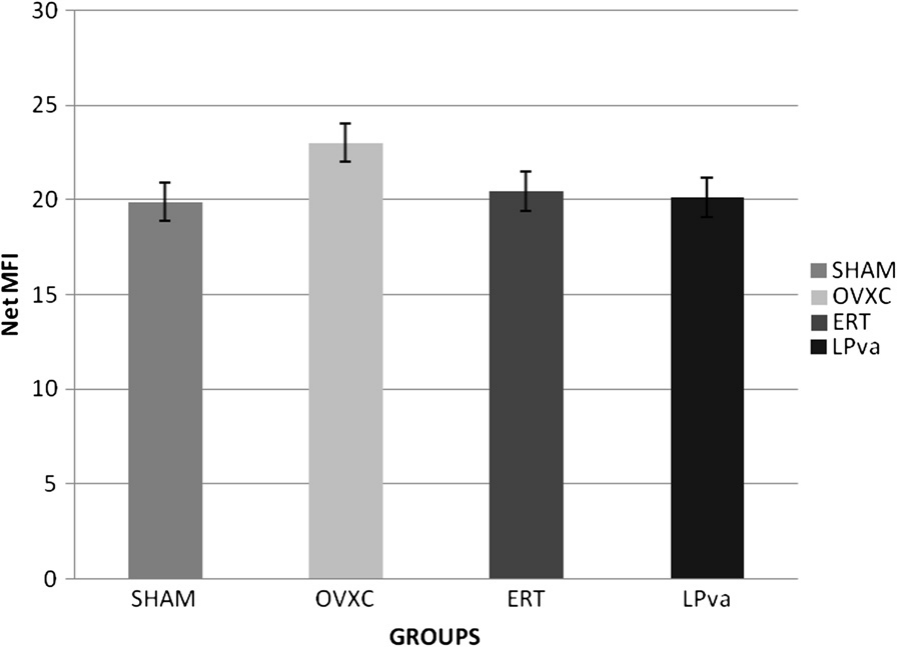
**MCSF Gene Expression.** Samples were normalized to GAPD MFI (MedianFluorescence Intensity). Error bars represent the standard deviations of the average responses. Values were expressed as mean ± SEM*. P < 0.05* is considered significant. ^a^ indicates significant difference compared to the Sham group. Sham (water vehicle), OVXC (water vehicle), LPva (*Labisia pumila var. Alata* 17.5 mg/kg/day), ERT (Premarin 64.5 μg/kg/day).

The BMP-2 gene expression of the femoral bones was significantly lower in the OVXC group compared to the other groups. Treatment with ERT was able to prevent the ovariectomy-induced reduction of BMP-2 gene expression, until it was at the same level as the Sham group. LPva supplementation was also able to prevent the ovariectomy-induced reduction in BMP-2 gene expression. There were no significant differences in the BMP-2 gene expression levels between the Sham, ERT and LPva groups (Figure 4).

## Discussion

Hormone replacement therapy (HRT/ERT) has been used for the treatment and prevention of postmenopausal osteoporosis, but it has been associated with serious side-effects (Ferguson, 2004) [36]. Multiple studies have reported that women who took HRT have slightly higher rates of thromboembolism, heart attack, breast cancer, ovarian cancer, stroke, and Alzheimer’s disease [8-10]. LPva has potential as an alternative to ERT for the treatment of postmenopausal osteoporosis. In terms of safety, several toxicity studies have confirmed that LPva is safe [37,38]. While, in terms of its action, LPva has demonstrated phytoestrogenic properties [20,21]. In an earlier study, LPva was found to reverse the bone biochemical marker changes due to ovariectomy [15]. Following that, a further study reported that LPva protected bone from osteoporotic changes due to estrogen deficiency. This was based on its ability to preserve the bone histomophometric parameters of ovariectomised rats [16]. These osteo-protective effects of LPva would be beneficial if they are accompanied by improvement in the bone strength, thus reducing the risk of fracture. This was confirmed by a biomechanical study which showed that supplementation of LPva in ovariectomised rats resulted in stronger femoral bone [17].

Several mechanisms of the bone protective effects of LPva were proposed. Other than acting as phytoestrogen, LPva may exert anti-inflammatory and anti-oxidant effects [17]. There were reports that inflammation may induce osteoporosis [39,40]. LPva may inhibit inflammation that may be responsible for osteoporosis by inhibiting tumor necrosis factor (TNF)-α production and down-regulating cyclooxygenase-2 expression [41].

Reactive oxygen species were shown to cause bone loss by stimulating osteoclast differentiation [42] and promoting osteoblast apoptosis [43]. LPva exhibited anti-oxidative properties as it contains flavanoids, ascorbic acid, beta-carotene, anthocyanin and phenolic compounds [44]. Beta-carotene was found to have the best correlation with anti-oxidative capacities of LP, followed by flavonoids, ascorbic acid, anthocyanin and phenolic content [45]. Flavonoids were confirmed by phytochemical screening to be present in our LPva extract. It is a potent free radical scavenger in oxidative stress-related diseases such as osteoporosis and rheumatism [46]. Other potent anti-oxidants such as vitamin E have also been shown to protect bone against osteoporosis [14]. Therefore, the anti-oxidative and anti-inflammatory properties of LPva extract may have contributed to the effectiveness of this medicinal plant in treating osteoporosis.

In the final pathway for pathogenesis of osteoporosis, there will be an imbalance between bone formation by osteoblast and bone formation by osteoclasts with the latter getting the upper hand. RANKL/OPG system, MCSF and BMP-2 played an important role in the regulation of the osteoclastic and osteoblastic activities. Therefore, their gene expressions were measured in this study to better understand the mechanism of LPva. To the best of our knowledge, this is the first report on the molecular mechanism of LPv in preventing bone loss due to estrogen-deficient osteoporosis.

The function of OPG is to block the binding of RANKL to RANK receptors on committed pre-osteclastic cells [47]. Therefore, OPG is a potent anti-osteoclastogenic factor. Estrogen is known to stimulate production of OPG, while, estrogen deficiency leads to down-regulation of OPG [48]. As expected, in the present study, the OPG gene expression of the ovariectomised control group was found to be down-regulated. Both the LPva supplementation and ERT were able to revert back the OPG gene expression to sham levels. This study has shown that osteo-protective mechanism of LPva may be similar to ERT i.e. via stimulation of OPG production.

Estrogen deficiency led to up-regulation of pro-inflammatory cytokines such as TNF-α and interleukins [49,50]. TNF is an important cofactor of bone resorption as it supports osteoclasts activation mediated by RANKL and c-Fms/MCSF. RANKL is a membrane-bound molecule of TNF ligand family which promotes osteoclasts formation [51]. In the present study, LPva may share similar mechanisms with ERT to protect bone as both were able to down-regulate the RANKL gene expression of ovariectomised rats.

This suggested a novel regulation of OPG and RANKL by LPva, which may help us to understand the mechanism of protection against estrogen-deficient bone loss. Interestingly, phytoestrogens such as genistein were also able to enhance osteoblastic OPG production through ER-dependent mechanisms and concurrently suppress RANKL gene expression which is associated with an inhibition of osteoclastogenesis [52-55]. Therefore, the phytoestrogenic element in LPva could be the reason for these novel findings.

We did not find any significant change in the MCSF gene expression after ovariectomy. An *in vitro* study of human endometrial stromal cells found that MCSF production was dose-dependently enhanced by the addition of sex hormone [56]. However, in this study, both ERT and LPva did not produce any significant changes in the MCSF gene expression. This meant that M-CSF was not affected in this model of osteoporosis.

BMP-2 plays an important role in bone repair and regeneration [31]. The BMP-2 gene expressions in the femora of ovariectomised rats were significantly increased by both LPva and ERT until they were similar to the sham level. Similar to our findings, Zhou *et. al*[57] also found that estrogen was able to activate BMP-2 gene transcription. Estrogen has been shown to stimulate the differentiation and activity of osteoblasts [58,59] and increase bone formation and bone mass in animal models [60,61]. Based on our results, the increased BMP-2 in LPva group was probably contributed by the phytoestrogenic effects of LPva.

## Conclusions

As a conclusion, LPva is comparable to ERT in regulating OPG, RANKL and BMP-2 gene expressions of ovariectomised rats. Therefore, LPva has potential as an alternative to ERT for the treatment and prevention of estrogen-deficient osteoporosis. LPva may also be taken as supplements by postmenopausal women who are uncomfortable with the risk of serious side-effects with ERT.

## Competing interests

The authors declare to have no conflict of interest whatsoever. The authors alone are responsible for the content and writing of this paper.

## Authors’ contributions

ANS, INS and INM designed the study. SNF carried out the study and collected the samples. NM and NM participated in the statistical analysis. SNF and ANS drafted the manuscript. INS, INM, NM and NM read and edited the manuscript. All authors approved the final manuscript.

## Pre-publication history

The pre-publication history for this paper can be accessed here:

http://www.biomedcentral.com/1472-6882/13/217/prepub
